# Supplementary material for: Effects of a Digital, Person-Centered, Photo-Activity Intervention on the Social Interaction of Nursing Home Residents with Dementia, Their Informal Carers and Formal Carers: An Explorative Randomized Controlled Trial
Source: Behav Sci (Basel). 2025 Jul 24;15(8):1008. doi: 10.3390/bs15081008 (PMC12383087; doi:10.3390/bs15081008)
Supplement: Supplementary file 1 [file behavsci-15-01008-s001.zip › behavsci-3720824-supplementary.pdf]

Table 1 | CONSORT 2025 checklist of information to include when reporting a randomised trial

Section/topic	No	CONSORT 2025 checklist item description	Section and Line in Manuscript
**Title and abstract**			
Title and structured abstract	1a	Identification as a randomised trial	Title page; 1-5
	1b	Structured summary of the trial design, methods, results, and conclusions	Title page; 24-40
**Open science**			
Trial registration	2	Name of trial registry, identifying number (with URL) and date of registration	Materials and Methods; 198-200
Protocol and statistical analysis plan	3	Where the trial protocol and statistical analysis plan can be accessed	Materials and Methods; 190-192
Data sharing	4	Where and how the individual de-identified participant data (including data dictionary), statistical code and any other materials can be accessed	Materials and Methods; 190-192
Funding and conflicts of interest	5a	Sources of funding and other support (eg, supply of drugs), and role of funders in the design, conduct, analysis and reporting of the trial	Funding; 952-959
	5b	Financial and other conflicts of interest of the manuscript authors	Conflicts of Interest; 974
**Introduction**			
Background and rationale	6	Scientific background and rationale	Introduction; 44-138
Objectives	7	Specific objectives related to benefits and harms	Introduction; 121-171
**Methods**			
Patient and public involvement	8	Details of patient or public involvement in the design, conduct and reporting of the trial	Materials and Methods; 174-183
Trial design	9	Description of trial design including type of trial (eg, parallel group, crossover), allocation ratio, and framework (eg, superiority, equivalence, non-inferiority, exploratory)	Materials and Methods; 174-192
Changes to trial protocol	10	Important changes to the trial after it commenced including any outcomes or analyses that were not prespecified, with reason	n/a
Trial setting	11	Settings (eg, community, hospital) and locations (eg, countries, sites) where the trial was conducted	Materials and Methods; 202-203
Eligibility criteria	12a	Eligibility criteria for participants	Materials and Methods; 217-223
	12b	If applicable, eligibility criteria for sites and for individuals delivering the interventions (eg, surgeons, physiotherapists)	Materials and Methods; 224-237
Intervention and comparator	13	Intervention and comparator with sufficient details to allow replication. If relevant, where additional materials describing the intervention and comparator (eg, intervention manual) can be accessed	Materials and Methods; 239-264; 384-443
Outcomes	14	Prespecified primary and secondary outcomes, including the specific measurement variable (eg, systolic blood pressure), analysis metric (eg, change from baseline, final value, time to event), method of aggregation (eg, median, proportion), and time point for each outcome	Materials and Methods; 285-382
Harms	15	How harms were defined and assessed (eg, systematically, non-systematically)	Materials and Methods; 190-192
Sample size	16a	How sample size was determined, including all assumptions supporting the sample size calculation	Materials and Methods; 204-210
	16b	Explanation of any interim analyses and stopping guidelines	Materials and Methods; 190-192
Randomisation:			
Sequence generation	17a	Who generated the random allocation sequence and the method used	Materials and Methods; 178-184
	17b	Type of randomisation and details of any restriction (eg, stratification, blocking and block size)	Materials and Methods; 178-184
Allocation concealment mechanism	18	Mechanism used to implement the random allocation sequence (eg, central computer/telephone; sequentially numbered, opaque, sealed containers), describing any steps to conceal the sequence until interventions were assigned	Materials and Methods; 178-184
Implementation	19	Whether the personnel who enrolled and those who assigned participants to the interventions had access to the random allocation sequence	Materials and Methods; 178-184
Blinding	20a	Who was blinded after assignment to interventions (eg, participants, care providers, outcome assessors, data analysts)	Materials and Methods; 224-233
	20b	If blinded, how blinding was achieved and description of the similarity of interventions	Materials and Methods; 224-233
Statistical methods	21a	Statistical methods used to compare groups for primary and secondary outcomes, including harms	Materials and Methods; 444-475
	21b	Definition of who is included in each analysis (eg, all randomised participants), and in which group	Materials and Methods; 444-475

	21c	How missing data were handled in the analysis	Materials and Methods; 444-475
	21d	Methods for any additional analyses (eg, subgroup and sensitivity analyses), distinguishing prespecified from post hoc	Materials and Methods; 444-475
**Results**			
Participant flow, including flow diagram	22a	For each group, the numbers of participants who were randomly assigned, received intended intervention, and were analysed for the primary outcome	Results; 476-480
	22b	For each group, losses and exclusions after randomisation, together with reasons	Results; 476-480
Recruitment	23a	Dates defining the periods of recruitment and follow-up for outcomes of benefits and harms	Results; 480- 483
	23b	If relevant, why the trial ended or was stopped	n/a
Intervention and comparator delivery	24a	Intervention and comparator as they were actually administered (eg, where appropriate, who delivered the intervention/comparator, how participants adhered, whether they were delivered as intended (fidelity))	Results; 481-483
	24b	Concomitant care received during the trial for each group	Materials and Methods; 435-440
Baseline data	25	A table showing baseline demographic and clinical characteristics for each group	Results (Table 2); 545
Numbers analysed, outcomes and estimation	26	For each primary and secondary outcome, by group:<br>the number of participants included in the analysis<br>the number of participants with available data at the outcome time point<br>result for each group, and the estimated effect size and its precision (such as 95% confidence interval) for binary outcomes, presentation of both absolute and relative effect size	Results; 500-665
Harms	27	All harms or unintended events in each group	Results; 599-609
Ancillary analyses	28	Any other analyses performed, including subgroup and sensitivity analyses, distinguishing pre-specified from post hoc	See Supplementary Materials (S3-S6)
**Discussion**			
Interpretation	29	Interpretation consistent with results, balancing benefits and harms, and considering other relevant evidence	Discussion; 668-809
Limitations	30	Trial limitations, addressing sources of potential bias, imprecision, generalisability, and, if relevant, multiplicity of analyses	Discussion; 811-913

TIDieR
Template for Intervention Description and Replication

The TIDieR (Template for Intervention Description and Replication) Checklist*:

Information to include when describing an intervention and the location of the information

Item number	Item	Where located **	
		Primary paper (line number)	Other † (details)
	**BRIEF NAME**		
**1.**	Provide the name or a phrase that describes the intervention.	121-124	______________
	**WHY**		
**2.**	Describe any rationale, theory, or goal of the elements essential to the intervention.	101-138	______________
	**WHAT**		
**3.**	Materials: Describe any physical or informational materials used in the intervention, including those provided to participants or used in intervention delivery or in training of intervention providers. Provide information on where the materials can be accessed (e.g. online appendix, URL).	239-256; 384-443	Tan et al., 2022 (Protocol paper)
**4.**	Procedures: Describe each of the procedures, activities, and/or processes used in the intervention, including any enabling or support activities.	384-443	
	**WHO PROVIDED**		
**5.**	For each category of intervention provider (e.g. psychologist, nursing assistant), describe their expertise, background and any specific training given.	384-443	
	**HOW**		
**6.**	Describe the modes of delivery (e.g. face-to-face or by some other mechanism, such as internet or telephone) of the intervention and whether it was provided individually or in a group.	384-443	
	**WHERE**		

**7.**	Describe the type(s) of location(s) where the intervention occurred, including any necessary infrastructure or relevant features.	385-389	
	**WHEN and HOW MUCH**		
**8.**	Describe the number of times the intervention was delivered and over what period of time including the number of sessions, their schedule, and their duration, intensity or dose.	406-421	______________
	**TAILORING**		
**9.**	If the intervention was planned to be personalised, titrated or adapted, then describe what, why, when, and how.	407-418	______________
	**MODIFICATIONS**		
**10.**[‡]	If the intervention was modified during the course of the study, describe the changes (what, why, when, and how).	433-435	
	**HOW WELL**		
**11.**	Planned: If intervention adherence or fidelity was assessed, describe how and by whom, and if any strategies were used to maintain or improve fidelity, describe them.	______________	Tan et al. 2025 (Process evaluation paper)
**12.**[‡]	Actual: If intervention adherence or fidelity was assessed, describe the extent to which the intervention was delivered as planned.	______________	Tan et al. 2025 (Process evaluation paper)

Tan, J. R. O., Boersma, P., Ettema, T. P., Aëgerter, L., Gobbens, R., Stek, M. L., & Dröes, R.-M. (2022). Known in the nursing home: development and evaluation of a digital person-centered artistic photo-activity intervention to promote social interaction between residents with dementia, and their formal and informal carers. *BMC geriatrics, 22*(1), 1-15.

Tan, J. R. O., Neal, D. P., Vilmen, M., Boersma, P., Ettema, T. P., Gobbens, R. J., . . . Dröes, R.-M. (2025). A Digital Photo Activity Intervention for Nursing Home Residents With Dementia and Their Carers: Mixed Methods Process Evaluation. *JMIR formative research, 9*, e56586.

Table S1: ANCOVA interaction effects between group and dementia severity for resident, formal carer and informal carer outcomes (ranges are shown per scale or sub-scale, with the positive or desirable score underlined).

**Residents**		**Pre-test (T0)**				**Post-test (T1)**				**ANCOVA**				
		**Experimental (n=38)**		**Control (n=39)**		**Experimental (n=30)**		**Control (n=32)**		**Experimental adjM (SE)**	**Control adjM (SE)**		**p**	**Effect Size Partial Eta²**
NPI-Q (10)	**GDS**	**M**	**(SD)**	**M**	**(SD)**	**M**	**(SD)**	**M**	**(SD)**					
**Total Severity Score** (range : <u>0</u>-30)	Low	2.50	(3.01)	3.36	(4.86)	3.05	(4.99)	2.19	(2.87)	3.60(.70)	2.20(.66)	GroupxGDS	.198	.029
	High	4.08	(3.58)	5.07	(4.20)	3.36	(3.93)	4.45	(4.53)	3.09(.91)	3.77(.91)			
		**Pre-test (T0)**				**Post-test (T1)**				**ANCOVA**				
		**Experimental (n=35)**		**Control (n=35)**		**Experimental (n=35)**		**Control (n=35)**		**Experimental adjM (SE)**	**Control adjM (SE)**		**p**	**Effect Size Partial Eta²**
SFAS Mean [a] (range: 1-<u>5</u>)	**GDS**	**M**	**(SD)**	**M**	**(SD)**	**M**	**(SD)**	**M**	**(SD)**					
	Low	3.55	(.59)	3.35	(.77)	3.84	(.64)	3.97	(.55)	3.80(.13)	4.02(.13)	GroupxGDS	.051	.057
	High	3.60	(.62)	3.42	(.37)	4.05	(.66)	3.60	(.86)	3.00(.17)	3.63(.17)			
		**Pre-test (T0)**				**Post-test (T1)**				**ANCOVA**				
		**Experimental (n=32)**		**Control (n=32)**		**Experimental (n=25)**		**Control (n=23)**		**Experimental adjM (SE)**	**Control adjM (SE)**		**p**	**Effect Size Partial Eta²**
Feeling Known Questions (Resident)	**GDS**	**M**	**(SD)**	**M**	**(SD)**	**M**	**(SD)**	**M**	**(SD)**					
To what extent do you feel you are known as a person here in the nursing home?	Low	6.77	(2.25)	6.65	(2.68)	7.76	(1.35)	7.14	(2.25)	7.74(.33)	7.46(.37)	GroupxGDS	.816	.001
(range: 0-<u>10</u>)	High	7.70	(1.06)	6.75	(2.77)	8.13	(.84)	7.44	(1.81)	7.78(.49)	7.31(.46)			
		**Pre-test (T0)**				**Post-test (T1)**				**ANCOVA**				
		**Experimental (n=34)**		**Control (n=34)**		**Experimental (n=27)**		**Control (n=26)**		**Experimental adjM (SE)**	**Control adjM (SE)**		**p**	**Effect Size Partial Eta²**

	GDS	M	(SD)	M	(SD)	M	(SD)	M	(SD)					
How satisfied are you with staying in the nursing home? (range: 0-10)	Low	7.91	(1.66)	7.05	(2.64)	7.67	(1.65)	7.38	(2.13)	7.31(.33)	7.85(.35)	GroupxGDS	.099	.056
	High	7.42	(1.62)	7.00	(2.80)	8.67	(1.12)	7.90	(1.60)	8.62(.45)	7.83(.43)			
		Pre-test (T0)				Post-test (T1)				ANCOVA				
		Experimental (n=38)		Control (n=39)		Experimental (n=30)		Control (n=32)		Experimental adjM (SE)	Control adjM (SE)		p	Effect Size Partial Eta^2
Qualidem subscales	GDS	M	(SD)	M	(SD)	M	(SD)	M	(SD)					
Care Relationship (range: 0-21)	Low	15.46	(4.17)	15.32	(4.10)	16.32	(3.67)	15.19	(4.78)	15.55(.70)	14.69(.67)	GroupxGDS	.241	.024
	High	15.25	(4.65)	12.93	(4.84)	15.18	(4.81)	14.36	(5.63)	15.38(.92)	16.44(.95)			
Positive Affect (range: 0-18)	Low	14.23	(3.83)	14.76	(2.85)	15.32	(3.70)	13.71	(3.91)	14.81(.54)	13.74(.51)	GroupxGDS	.538	.007
	High	14.17	(3.95)	12.93	(4.84)	15.00	(3.85)	13.64	(3.91)	15.66(.71)	13.81(.71)			
Negative Affect (range: 0-9)	Low	5.35	(2.71)	5.84	(2.08)	6.16	(2.34)	5.71	(1.88)	6.04(.31)	5.51(.30)	GroupxGDS	.364	.014
	High	5.42	(2.94)	4.71	(2.43)	6.00	(2.93)	5.55	(2.02)	6.01(.41)	6.13(.41)			
Restless Tense Behaviour (range: 0-9)	Low	5.23	(2.61)	5.44	(2.70)	5.74	(3.0)	5.43	(2.79)	5.39(.47)	5.36(.45)	GroupxGDS	.302	.019
	High	5.42	(2.15)	3.93	(2.59)	6.36	(2.62)	4.27	(3.20)	6.27(.62)	5.11(.62)			
Positive Self-Esteem (range: 0-9)	Low	6.62	(2.04)	6.6	(1.92)	7.37	(1.86)	6.71	(1.71)	7.03(.28)	6.59(.26)	GroupxGDS	.236	.025
	High	6.00	(2.86)	5.86	(2.03)	6.64	(2.50)	6.82	(2.60)	6.98(.36)	7.30(.36)			
Social Relationships (range: 0-18)	Low	10.92	(2.17)	10.44	(2.79)	11.53	(3.01)	10.19	(2.66)	11.15(.52)	10.45(.49)	GroupxGDS	.758	.002
	High	9.75	(2.73)	10.57	(2.82)	11.45	(2.25)	11.09	(2.66)	11.89(.68)	10.81(.68)			
Social Isolation (range: 0-9)	Low	7.04	(2.09)	6.44	(2.08)	7.53	(1.71)	6.33	(1.80)	7.10(.30)	6.35(.28)	GroupxGDS	.282	.020
	High	6.5	(2.36)	5.43	(2.44)	6.91	(1.70)	6.36	(2.42)	6.99(.39)	6.99(.40)			
Feeling at Home (range: 0-12)	Low	8.62	(3.01)	8.76	(3.37)	9.16	(3.50)	8.48	(3.59)	8.70(.45)	8.53(.43)	GroupxGDS	.876	.000
	High	8.75	(2.67)	8.29	(3.54)	8.64	(3.61)	8.55	(2.58)	8.93(.60)	8.925(.60)			

Have Something to Do (range: 0-6)	Low	3.35	(1.74)	3.16	(1.70)	3.53	(1.87)	3.14	(1.53)	3.39(.29)	3.13(.27)	GroupxGDS	.866	.001
	High	2.83	(1.53)	2.64	(1.74)	3.36	(1.57)	3.09	(1.81)	3.43(.38)	3.28(.38)			
**Formal Carers**		**Pre-test (T0)**				**Post-test (T1)**				**ANCOVA**				
		**Experimental (n=23)**		**Control (n=28)**		**Experimental (n=19)**		**Control (n=22)**		**Experimental adjM (SE)**	**Control adjM (SE)**		**p**	**Effect Size Partial Eta²**
IRI	**GDS**	**M**	**(SD)**	**M**	**(SD)**	**M**	**(SD)**	**M**	**(SD)**					
Global Empathy (range: 0-112)	Low	62.07	9.18	59.00	9.92	58.50	7.32	62.65	8.56	57.95(1.73)	64.00(1.48)	Groupx TotalBaselineADQ	.663	.005
	High	67.75	5.65	61.67	8.22	61.43	4.50	61.40	10.14	58.29(2.34)	62.50(2.67)			
**Informal Carers**		**Pre-test (T0)**				**Post-test (T1)**				**ANCOVA**				
		**Experimental (n=37)**		**Control (n=37)**		**Experimental (n=32)**		**Control (n=28)**		**Experimental adjM (SE)**	**Control adjM (SE)**		**p**	**Effect Size Partial Eta²**
SSCQ	**GDS**	**M**	**(SD)**	**M**	**(SD)**	**M**	**(SD)**	**M**	**(SD)**					
Feeling of Competence (range: 5-35)	Low	27.63	(5.640)	26.84	(5.632)	27.60	(3.803)	27.83	(5.382)	27.716(.717)	28.289(.759)	GroupxGDS	.420	.012
	High	30.77	(3.678)	27.25	(4.434)	29.58	(3.630)	29.50	(2.068)	28.162(.954)	30.153(1.019)			
		**Pre-test (T0)**				**Post-test (T1)**				**ANCOVA**				
		**Experimental (n=37)**		**Control (n=37)**		**Experimental (n=37)**		**Control (n=37)**		**Experimental adjM (SE)**	**Control adjM (SE)**		**p**	**Effect Size Partial Eta²**
Feeling Known Questions (Informal Carer)	**GDS**	**M**	**(SD)**	**M**	**(SD)**	**M**	**(SD)**	**M**	**(SD)**					
To what extent do you feel seen as the family carer of your loved one in the nursing home? (range: 1-10)	Low	8.04	(1.81)	8.56	(1.26)	8.40	(1.96)	8.22	(1.52)	8.76(.18)	8.20(.18)	GroupxGDS	.086	.053
	High	8.85	(.99)	8.42	(1.44)	8.58	(.79)	8.70	(1.64)	8.25(.23)	8.42(.25)			
		**Pre-test (T0)**				**Post-test (T1)**				**ANCOVA**				
		**Experimental (n=37)**		**Control (n=37)**		**Experimental (n=37)**		**Control (n=37)**		**Experimental adjM (SE)**	**Control adjM (SE)**		**p**	**Effect Size Partial Eta²**
		**M**	**(SD)**	**M**	**(SD)**	**M**	**(SD)**	**M**	**(SD)**					

How satisfied are you with the stay of your loved one with dementia in the nursing home? (range: 1-<u>10</u>)	Low	8.46	(1.25)	8.40	(1.44)	8.65	(1.35)	8.17	(2.07)	8.70(.17)	8.56(.18)	GroupxGDS	.624	.004
	High	9.00	(1.00)	8.92	(1.08)	8.83	(.94)	9.20	(1.14)	8.60(.22)	8.66(.24)			
		**Pre-test (T0)**				**Post-test (T1)**				**ANCOVA**				
		**Experimental (n=37)**		**Control (n=37)**		**Experimental (n=37)**		**Control (n=37)**		**Experimental adjM (SE)**	**Control adjM (SE)**		**p**	**Effect Size Partial Eta²**
		**M**	**(SD)**	**M**	**(SD)**	**M**	**(SD)**	**M**	**(SD)**					
To what extent do you feel that your loved one with dementia is known in the nursing home? (range: 1-<u>10</u>)	Low	8.63	(1.35)	8.44	(1.33)	8.70	(1.22)	8.28	(1.87)	8.78(.20)	8.54(.21)	GroupxGDS	.985	.000
	High	9.08	(1.04)	8.92	(8.83)	8.75	(1.06)	8.60	(1.35)	8.50(.26)	8.26(.28)			

[a]SFAS Mean: Pre-test: Mean of all Before SFAS Week 1 and 4; Post-test: Mean of all After SFAS Week 1 and 4

Table S2: ANCOVA interaction effects between group and Hours (of visit) of informal carers for resident, formal carer and informal carer outcomes (ranges are shown per scale or sub-scale, with the positive or desirable score underlined).

		Pre-test (T0)				Post-test (T1)				ANCOVA				
		Experimental (n=38)		Control (n=35)		Experimental (n=30)		Control (n=28)		Experimental adjM (SE)	Control a djM (SE)		p	Effect Size Partial Eta^2
Qualidem subscales	**Hours Visit**	**M**	**(SD)**	**M**	**(SD)**	**M**	**(SD)**	**M**	**(SD)**					
Care Relationship (0-21)	Low	16.15	(4.25)	15.05	(3.85)	16.71	(3.06)	16.29	(4.22)	16.05(0.70)	16.51(0.70)	GroupxHoursVisit	.302	.020
	High	14.56	(4.25)	15.13	(4.31)	14.85	(5.06)	14.45	(5.17)	15.51(0.80)	14.35(0.87)			
Positive Affect (0-18)	Low	15.50	(2.74)	15.55	(2.61)	15.88	(3.43)	14.88	(2.98)	15.14(0.57)	14.34(0.56)	GroupxHoursVisit	.571	.006
	High	12.78	(4.37)	13.87	(3.48)	14.31	(3.97)	12.91	(4.42)	15.26(0.65)	13.76(0.70)			
Negative Affect (0-9)	Low	6.15	(2.37)	5.50	(2.37)	7.06	(1.64)	5.47	(2.04)	6.54(0.33)	5.71(0.33)	GroupxHoursVisit	.204	.030
	High	4.50	(2.94)	5.80	(2.15)	4.85	(2.97)	6.09	(2.02)	5.56(0.38)	5.67(0.41)			
Restless Tense Behaviour (0-9)	Low	5.6	(2.034)	5.05	(2.72)	6.76	(2.46)	5.41	(2.90)	6.45(0.48)	5.71(0.48)	GroupxHoursVisit	.368	.015
	High	4.94	(2.856)	5.00	(2.98)	4.92	(3.04)	5.18	(2.89)	4.95(0.54)	5.17(0.59)			
Positive Self-Esteem (0-9)	Low	7.40	(1.76)	6.30	(1.87)	7.76	(1.25)	6.76	(1.71)	6.96(0.31)	7.00(0.30)	GroupxHoursVisit	.524	.008
	High	5.33	(2.40)	6.53	(2.33)	6.23	(2.68)	7.00	(2.72)	7.17(0.35)	6.77(0.37)			
Social Relationships (0-18)	Low	10.60	(2.56)	11.15	(2.28)	11.41	(2.60)	11.00	(2.57)	11.31(0.55)	10.83(0.55)	GroupxHoursVisit	.637	.004
	High	10.50	(2.26)	(9.53)	(3.14)	11.62	(2.96)	10.09	(2.47)	11.60(0.63)	10.54(0.69)			
Social Isolation (0-9)	Low	7.25	(1.86)	6.60	(1.79)	7.76	(1.52)	7.06	(1.71)	7.30(0.29)	7.07(0.29)	GroupxHoursVisit	.874	.000
	High	6.44	(2.43)	5.73	(2.37)	6.69	(1.80)	6.18	(1.94)	6.93(0.33)	6.60(0.36)			
Feeling at Home (0-12)	Low	9.60	(1.98)	8.70	(2.90)	10.41	(2.06)	8.47	(3.09)	9.62(0.47)	8.89(0.46)	GroupxHoursVisit	.144	.040
	High	7.61	(3.36)	8.93	(4.15)	7.080	(4.11)	9.36	(3.38)	8.01(0.54)	8.84(0.58)			
Have Something to Do (0-6)	Low	3.15	(1.73)	3.60	(1.60)	3.12	(1.87)	3.59	(1.58)	3.11(0.31)	3.34(0.31)	GroupxHoursVisit	.189	.032
	High	3.22	(1.67)	2.27	(1.62)	3.92	(1.50)	2.73	(1.56)	3.87(0.35)	3.187(0.39)			

	**Pre-test (T0)**					**Post-test (T1)**				**ANCOVA**				
		**Experimental (n=38)**		**Control (n=39)**		**Experimental (n=30)**		**Control (n=28)**		**Experimental adjM (SE)**	**Control adjM (SE)**		**p**	**Effect Size Partial Eta²**
NPI-Q **(10)**	**Hours Visit**	**M**	**(SD)**	**M**	**(SD)**	**M**	**(SD)**	**M**	**(SD)**					
Total Severity Score (range: 0-30)	Low	2.05	(1.99)	3.85	(5.32)	1.76	(2.36)	2.29	(2.87)	2.64(0.75)	1.77(0.74)	GroupxHoursVisit	.963	.000
	High	4.06	(4.02)	3.53	(3.89)	5.00	(6.04)	2.73	(4.38)	4.14(0.85)	3.20(0.91)			
	**Pre-test (T0)**					**Post-test (T1)**				**ANCOVA**				
	**Hours Visit**	**Experimental (n=37)**		**Control (n=37)**		**Experimental (n=32)**		**Control (n=28)**		**Experimental adjM (SE)**	**Control adjM (SE)**		**p**	**Effect Size Partial Eta²**
SSCQ		**M**	**(SD)**	**M**	**(SD)**	**M**	**(SD)**	**M**	**(SD)**					
Feeling of Competence (range: 7-35)	Low	29.37	(4.51)	28.20	(4.18)	28.78	(3.04)	28.12	(4.66)	28.19(0.75)	28.14(0.77)	GroupxHoursVisit	.091	.051
	High	28.06	(5.92)	25.53	(6.03)	27.80	(4.68)	28.91	(4.44)	27.43(0.85)	30.27(0.98)			
	**Pre-test (T0)**					**Post-test (T1)**				**ANCOVA**				
		**Experimental (n=35)**		**Control (n=32)**		**Experimental (n=35)**		**Control (n=32)**		**Experimental adjM (SE)**	**Control adjM (SE)**		**p**	**Effect Size Partial Eta²**
INTERACT	**Hours Visit**	**M**	**(SD)**	**M**	**(SD)**	**M**	**(SD)**	**M**	**(SD)**					
Mood (range: 0-24)	Low	20.89	(2.47)	19.16	(3.53)	20.47	(2.55)	17.67	(4.27)	19.54(0.78)	17.39(0.73)	GroupxHoursVisit	.360	.015
	High	18.12	(4.97)	15.62	(4.99)	18.07	(3.32)	16.36	(3.50)	18.44(0.83)	17.783(0.99)			
Speech (range: 0-20)	Low	16.95	(2.61)	16.74	(2.40)	16.65	(3.92)	15.56	(3.40)	15.77(0.92)	14.85(0.89)	GroupxHoursVisit	.320	.018
	High	15.25	(5.18)	12.23	(5.17)	14.00	(6.16)	13.55	(4.95)	14.44(1.00)	15.50(1.18)			
Relating to Person (range: 0-24)	Low	20.05	(2.61)	18.79	(1.81)	18.76	(3.09)	18.56	(2.28)	18.01(0.72)	18.50(0.67)	GroupxHoursVisit	.540	.007
	High	17.88	(3.86)	16.92	(2.96)	17.29	(3.45)	16.64	(3.70)	17.74(0.77)	17.32(0.87)			
Relating to Environment (range: 0-20)	Low	15.68	(3.45)	15.79	(2.62)	14.71	(3.92)	15.89	(3.36)	14.73(0.98)	15.90(0.96)	GroupxHoursVisit	.751	.002
	High	16.00	(3.56)	12.31	(5.523)	14.21	(4.19)	14.82	(4.88)	14.25(1.09)	14.73(1.25)			
Need for Prompting (range: 0-4)	Low	2.95	(0.97)	2.79	(1.08)	2.82	(1.19)	2.61	(1.09)	2.68(0.28)	2.54(0.27)	GroupxHoursVisit	.117	.044
	High	2.75	(1.34)	1.69	(1.44)	1.86	(1.46)	2.36	(1.29)	1.86(0.31)	2.70(0.37)			
Stimulation Level	Low	18.58	(1.87)	17.32	(2.06)	18.60	(1.94)	16.72	(2.78)	17.96(0.73)	16.62(0.67)	GroupxHoursVisit	.545	.007

(range: 0-20)														
	High	16.50	(3.65)	15.23	(3.94)	16.36	(3.43)	15.55	(3.93)	16.69(0.77)	16.27(0.90)			
Negative Interactions (range: 0-24)	Low	23.74	(0.56)	23.21	(1.27)	23.71	(0.47)	22.83	(1.92)	23.39(0.32)	22.88(0.30)	GroupxHoursVisit	.875	.000
	High	23.50	(1.10)	22.62	(1.50)	23.79	(0.58)	22.45	(2.30)	23.66(0.34)	23.04(0.41)			
	**Pre-test (T0)**					**Post-test (T1)**				**ANCOVA**				
	**Hours Visit**	**Experimental (n=35)**		**Control (n=35)**		**Experimental (n=35)**		**Control (n=31)**		**Experimental adjM (SE)**	**Control adjM (SE)**		**p**	**Effect Size Partial Eta^2**
SFAS Mean [a] **(range: 1-5)**		**M**	**(SD)**	**M**	**(SD)**	**M**	**(SD)**	**M**	**(SD)**					
	Low	3.68	(0.60)	3.32	(0.77)	4.04	(0.67)	3.95	(0.46)	3.96(0.12)	4.02(0.12)	GroupxHoursVisit	.592	.005
	High	3.43	(0.53)	3.51	(0.51)	3.77	(0.59)	4.01	(0.65)	3.79(0.13)	4.01(0.15)			
	**Pre-test (T0)**					**Post-test (T1)**				**ANCOVA**				
		**Experimental (n=32)**		**Control (n=28)**		**Experimental (n=25)**		**Control (n=19)**		**Experimental adjM (SE)**	**Control adjM (SE)**		**p**	**Effect Size Partial Eta^2**
Feeling Known Questions (Resident)	**Hours Visit**	**M**	**(SD)**	**M**	**(SD)**	**M**	**(SD)**	**M**	**(SD)**					
To what extent do you feel you are known as a person here in the nursing home?	Low	7.59	(1.81)	7.20	(2.40)	7.86	(1.17)	7.38	(2.29)	7.65(.37)	7.52(0.39)	GroupxHoursVisit	.434	.016
(range: 0-10)	High	8.56	(1.29)	6.38	(3.02)	7.91	(1.30)	7.17	(2.14)	8.01(0.42)	7.18(0.57)			
	**Pre-test (T0)**					**Post-test (T1)**				**ANCOVA**				
		**Experimental (n=34)**		**Control (n=30)**		**Experimental (n=27)**		**Control (n=22)**		**Experimental adjM (SE)**	**Control adjM (SE)**		**p**	**Effect Size Partial Eta^2**
		**M**	**(SD)**	**M**	**(SD)**	**M**	**(SD)**	**M**	**(SD)**					
How satisfied are you with staying in the nursing home? (range: 0-10)	Low	8.11	(1.45)	7.38	(2.25)	8.20	(1.52)	7.36	(2.21)	7.80(.37)	7.55(0.38)	GroupxHoursVisit	.486	.011
	High	7.31	(1.78)	7.00	(3.16)	7.75	(1.60)	7.88	(1.46)	7.84(0.41)	8.17(0.50)			

	**Pre-test (T0)**					**Post-test (T1)**				**ANCOVA**				
		**Experimental (n=37)**		**Control (n=37)**		**Experimental (n=32)**		**Control (n=28)**		**Experimental adjM (SE)**	**Control adjM (SE)**		**p**	**Effect Size Partial Eta²**
Feeling Known Questions (Informal Carer)	**Hours Visit**	**M**	**(SD)**	**M**	**(SD)**	**M**	**(SD)**	**M**	**(SD)**					
To what extent do you feel seen as the family carer of your loved one in the nursing home? (range: 0-10)	Low	8.04	(1.81)	8.56	(1.26)	8.56	(2.04)	8.76	(1.35)	8.69(.19)	8.431(.19)	GroupxHoursVisit	.787	.001
	High	8.85	(0.99)	8.42	(1.44)	8.36	(0.84)	7.82	(1.72)	8.41(0.21)	8.04(0.24)			
	**Pre-test (T0)**					**Post-test (T1)**				**ANCOVA**				
		**Experimental (n=37)**		**Control (n=37)**		**Experimental (n=32)**		**Control (n=28)**		**Experimental adjM (SE)**	**Control adjM (SE)**			
	**Hours Visit**	**M**	**(SD)**	**M**	**(SD)**	**M**	**(SD)**	**M**	**(SD)**				**p**	**Effect Size Partial Eta²**
How satisfied are you with the stay of your loved one with dementia in the nursing home? (range: 0-10)	Low	8.89	(1.20)	9.05	(1.23)	8.94	(1.26)	9.00	(1.37)	8.80(0.17)	8.68(0.18)	GroupxHours	.798	.001
	High	8.39	(1.15)	8.00	(1.28)	8.43	(1.09)	7.82	(2.27)	8.49(0.20)	8.47(0.23)			
	**Pre-test (T0)**					**Post-test (T1)**				**ANCOVA**				
		**Experimental (n=37)**		**Control (n=37)**		**Experimental (n=32)**		**Control (n=28)**						
	**Hours Visit**	**M**	**(SD)**	**M**	**(SD)**	**M**	**(SD)**	**M**	**(SD)**	**Experimental adjM (SE)**	**Control adjM (SE)**		**p**	**Effect Size Partial Eta²**
To what extent do you feel that your loved one with dementia is known in the nursing home? (range: 0-10)	Low	9.00	(1.20)	9.10	(0.91)	9.00	(1.24)	8.94	(1.09)	8.82(0.21)	8.61(0.22)	GroupxHoursVisit	.802	.001
	High	8.56	(1.29)	7.94	(1.48)	8.36	(0.93)	7.55	(2.12)	8.50(0.24)	8.17(0.28)			

[a]SFAS Mean: Pre-test: Mean of all Before SFAS Week 1 and 4; Post-test: Mean of all After SFAS Week 1 and 4

Table S3: Mixed model for repeated measures (MMRM) interaction effects between group, dementia severity, and time (ranges are shown per scale or sub-scale, with the positive or desirable score underlined).

				**Means**					**Effect Estimate**	***p-value***	**Effect Size**
Qualidem		**T0**		**T1**		**T2**			(GroupxGDSxTime)		*Cohen's d*
	**GDS**	**Exp**	**Control**	**Exp**	**Control**	**Exp**	**Control**				
Care relationships (range: 0-21)	Low	16.88	16.88	17.15	16.35	16.56	15.94	GroupXGDSxT1	-1.57	.306	-0.36
	High	15.12	15.12	15.71	16.48	15.98	16.13	GroupxGDSxT2	-.77	.647	-.18
Positive Affect (range: 0-18)	Low	15.23	15.23	15.21	14.19	14.95	13.75	GroupXGDSxT1	.81	.513	.23
	High	14.86	14.86	15.83	13.99	15.93	14.34	GroupxGDSxT2	.39	.775	.11
Negative Affect (range: 0-9)	Low	6.50	6.50	6.94	6.44	6.68	6.65	GroupXGDSxT1	-.64	.381	-.26
	High	5.78	5.78	6.47	6.60	6.14	7.25	GroupxGDSxT2	-1.14	.192	-.46
Restless Tense Behaviour (range: 0-9)	Low	5.47	5.47	5.44	5.42	5.35	5.71	GroupXGDSxT1	1.28	.229	.50
	High	4.58	4.58	5.88	4.59	4.81	4.90	GroupxGDSxT2	.27	.768	.11
Positive Self-Esteem (range: 0-9)	Low	7.30	7.30	7.70	7.33	7.22	7.12	GroupXGDSxT1	-.73	.249	-.34
	High	6.48	6.48	7.08	7.44	7.45	7.49	GroupxGDSxT2	-.14	.854	-.07
Social Relationships (range: 0-18)	Low	10.82	10.82	11.06	10.41	10.05	10.17	GroupXGDSxT1	.51	.665	.20
	High	10.20	10.2	11.57	10.41	11.02	9.35	GroupxGDSxT2	1.80	.056	.70
Social Isolation (range: 0-9)	Low	6.21	6.21	6.57	5.86	6.37	5.80	GroupXGDSxT1	-.58	.383	-.26
	High	5.31	5.31	6.10	5.96	6.10	5.93	GroupxGDSxT2	-.40	.667	-.18
Feeling at Home (range: 0-12)	Low	8.72	8.72	8.46	8.39	8.35	8.09	GroupXGDSxT1	-.01	.996	.00
	High	8.36	8.36	8.48	8.43	9.13	8.96	GroupxGDSxT2	-.08	.936	-.03
Have Something to Do (range: 0-6)	Low	2.96	2.96	3.03	2.87	3.26	2.53	GroupXGDSxT1	.002	.997	.00
	High	2.42	2.42	2.83	2.66	2.84	2.76	GroupxGDSxT2	-.66	.339	-.39
NPI-Q **(**10)		**T0**		**T1**		**T2**			**Effect Estimate**	***p-value***	**Effect Size**
	**GDS**	**Exp**	**Control**	**Exp**	**Control**	**Exp**	**Control**		(GroupxGDSxTime)		*Cohen's d*
Total Severity Score (range : 0-30)	Low	3.278	3.278	3.701	2.154	3.606	2.636	GroupxGDSxT1	-2.412	.123	-.60
	High	5.137	5.137	4.157	5.022	4.238	4.304	GroupxGDSxT2	-1.036	.588	-.26

Short Sense of Competence		**T0**		**T1**		**T2**			**Effect Estimate**	***p-value***	**Effect Size**
	**GDS**	**Exp**	**Control**	**Exp**	**Control**	**Exp**	**Control**		(GroupxGDSxTime)		*Cohen's d*
Total Sense of Competence Score (range: 5-35)	Low	28.72	28.72	28.61	29.15	29.34	29.74	GroupxGDSxT1	-1.32	.444	-.25
	High	30.12	30.12	29.61	31.47	29.31	31.31	GroupxGDSxT2	-1.60	.370	-.30
Questions for Resident		**T0**		**T1**		**T2**			**Effect Estimate**	***p-value***	**Effect Size**
	**GDS**	**Exp**	**Control**	**Exp**	**Control**	**Exp**	**Control**		(GroupxGDSxTime)		*Cohen's d*
Regarding feeling known as a person	Low	7.49	7.49	8.20	7.54	7.89	7.61	GroupxGDSxT1	-.50	.562	-.21
(range: 1-10)	High	7.87	7.87	8.16	8.00	8.14	8.07	GroupxGDSxT2	-.20	.824	-.09
Regarding feeling satisfied with nursing home care (range: 1-10)	Low	7.50	7.50	7.31	7.64	7.25	7.09	GroupxGDSxT1	.87	.282	.39
	High	7.36	7.36	8.36	7.81	7.26	7.32	GroupxGDSxT2	-.23	.751	-.10
Questions for Informal Carer		**T0**		**T1**		**T2**			**Effect Estimate**	***p-value***	**Effect Size**
	**GDS**	**Exp**	**Control**	**Exp**	**Control**	**Exp**	**Control**		(GroupxGDSxTime)		*Cohen's d*
Regarding feeling known as the informal carer	Low	8.51	8.51	8.84	8.29	8.57	8.54	GroupxGDSxT1	-.72	.087	-.50
(range: 1-10)	High	8.68	8.68	8.51	8.68	8.55	8.38	GroupxGDSxT2	.15	.737	.10
Regarding feeling satisfied with loved one's stay in nursing home	Low	8.46	8.46	8.46	8.29	8.45	8.11	GroupxGDSxT1	-.25	.541	-.20
(range: 1-10)	High	8.85	8.85	8.76	8.84	8.53	8.59	GroupxGDSxT2	-.39	.486	-.31
Regarding feeling that loved one is known in nursing home	Low	8.63	8.63	8.69	8.45	8.61	8.23	GroupxGDSxT1	.02	.965	.02
(range: 1-10)	High	8.92	8.92	8.69	8.43	8.54	8.64	GroupxGDSxT2	-.48	.319	-.37

Table S4: Mixed model for repeated measures (MMRM) interaction effects between group, hours visit of informal carer, and time (ranges are shown per scale or sub-scale, with the positive or

desirable score underlined).

				**Means**					**Effect Estimate**	***p-value***	**Effect Size**
Qualidem		**T0**		**T1**		**T2**			(GroupxHoursxTime)		*Cohen's d*
	**Hours Visit**	**Exp**	**Control**	**Exp**	**Control**	**Exp**	**Control**				
Care relationships (range: 0-21)	Low	15.823	15.823	16.36	16.79	15.70	15.87	GroupXHoursxT1	1.54	.286	.35
	High	15.83	15.83	15.97	14.86	16.27	14.72	GroupxHoursxT2	1.72	.318	.39
Positive Affect (range: 0-18)	Low	15.08	15.08	15.41	14.75	15.36	14.20	GroupXHoursxT1	.95	.437	.27
	High	15.078	15.08	15.44	13.83	15.18	13.67	GroupxHoursxT2	.35	.803	.10
Negative Affect (range: 0-9)	Low	6.19	6.19	7.12	6.35	6.64	6.98	GroupXHoursxT1	-.83	.25	-.34
	High	6.19	6.19	6.18	6.25	6.14	6.58	GroupxHoursxT2	-.10	.916	-.04
Restless Tense Behaviour (range: 0-9)	Low	5.00	5.00	6.18	5.30	5.68	5.45	GroupxHoursxT1	-1.24	.228	-.48
	High	5.00	5.00	4.50	4.86	4.08	5.18	GroupxHoursxT2	-1.34	.138	-.52
Positive Self-Esteem (range: 0-9)	Low	6.91	6.91	7.29	7.37	7.16	7.23	GroupXHoursxT1	.40	.534	.19
	High	6.91	6.91	7.46	7.14	7.31	7.23	GroupxHoursxT2	-.12	.875	-.06
Social Relationships (range: 0-18)	Low	10.43	10.43	10.90	10.43	10.27	10.11	GroupXHoursxT1	.55	.648	.21
	High	10.43	10.43	11.31	10.27	10.23	9.30	GroupxHoursxT2	.78	.425	.30
Social Isolation (range: 0-9)	Low	5.64	5.64	6.27	5.99	6.21	6.16	GroupXHoursxT1	.05	.932	.02
	High	5.64	5.64	6.04	5.71	5.89	5.49	GroupxHoursxT2	.36	.683	.16
Feeling at Home (range: 0-12)	Low	8.58	8.58	9.19	8.50	9.14	8.39	GroupXHoursxT1	-1.63	.100	-.52
	High	8.58	8.58	7.52	8.46	8.03	8.53	GroupxHoursxT2	-1.25	.238	-.40
Have Something to Do (range: 0-6)	Low	2.68	2.68	2.58	2.85	3.05	2.58	GroupXHoursxT1	.90	.171	.53
	High	2.68	2.68	3.28	2.65	2.99	2.43	GroupxHoursxT2	.09	.894	.06
NPI-Q (10)		**T0**		**T1**		**T2**			**Effect Estimate**	***p-value***	**Effect Size**

	**Hours Visit**	**Exp**	**Control**	**Exp**	**Control**	**Exp**	**Control**		(GroupxHoursxTime)		*Cohen's d*
Total Severity Score (range : 0-30)	Low	4.25	4.25	3.48	2.81	3.55	3.52	GroupXHoursxT1	.67	.671	.17
	High	4.25	4.25	5.07	3.73	4.90	2.97	GroupxHoursxT2	1.91	.331	.47
Short Sense of Competence		**T0**		**T1**		**T2**			**Effect Estimate**	***p-value***	**Effect Size**
	**Hours Visit**	**Exp**	**Control**	**Exp**	**Control**	**Exp**	**Control**		(Group HoursxTime)		*Cohen's d*
Total Sense of Competence Score (range: 5-35)	Low	29.60	29.560	29.55	29.434	30.20	29.84	GroupXHoursxT1	-2.86	.086	-.54
	High	29.60	29.60	29.09	31.83	29.10	32.03	GroupxHoursxT2	-3.29	.056	-.63
Questions for Resident		**T0**		**T1**		**T2**			**Effect Estmate**	***p-value***	**Effect Size**
	**Hours Visit**	**Exp**	**Control**	**Exp**	**Control**	**Exp**	**Control**		(GroupxHoursxTim e)		*Cohen's d*
Regarding feeling known as a person (range: 1-10)	Low	7.67	7.67	8.15	7.82	8.08	8.01	GroupXHoursxT1	.68	.440	.29
	High	7.67	7.67	8.36	7.36	7.97	7.04	GroupxHoursxT2	.86	.347	.37
Regarding feeling satisfied with nursing home care	Low	7.53	7.53	7.62	7.32	7.30	7.36	GroupXHoursxT1	-.65	.441	-.29
(range: 1-10)	High	7.53	7.53	7.87	8.22	7.35	6.79	GroupxHoursxT2	.61	.386	.28
**Questions for Informal Carer**		**T0**		**T1**		**T2**			**Effect Estimate**	***p-value***	**Effect Size**
	**Hours Visit**	**Exp**	**Control**	**Exp**	**Control**	**Exp**	**Control**		(GroupxHoursxTim e)		*Cohen's d*
Regarding feeling known as the informal carer	Low	8.59	8.59	8.87	8.60	8.61	8.45	GroupXHoursxT1	.08	.856	.05
(range: 1-10)	High	8.59	8.59	8.57	8.23	8.55	8.59	GroupxHoursxT2	-.20	.643	-.14
Regarding feeling satisfied with loved one's stay in nursing home	Low	8.68	8.68	8.79	8.67	8.60	8.44	GroupXHoursxT1	-.06	.886	-.04
(range: 1-10)	High	8.68	8.68	8.49	8.42	8.51	8.26	GroupxHoursxT2	.08	.883	.06
Regarding feeling that loved one is known in	Low	8.80	8.80	8.89	8.69	8.61	8.40	GroupXHoursxT1	.14	.754	.11

nursing home (range: 1-10)	High	8.80	8.80	8.60	8.25	8.67	8.52	GroupxHoursxT2	-.05	.909	-.04

Tan, J. R. O., Neal, D. P., Vilmen, M., Boersma, P., Ettema, T. P., Gobbens, R. J., . . . Dröes, R.-M. (2025). A Digital Photo Activity Intervention for Nursing Home Residents With Dementia and Their Carers: Mixed Methods Process Evaluation. *JMIR formative research, 9*, e56586.
